# Iridotomy

**Published:** 1875-10

**Authors:** 


IRIDOTOMY. By A. W. Calhoun, M.D., Professor of Diseases of Eye and Ear, Atlanta Medical College. Pamphlet: pp. 8.

In this pamphlet the author explains the terms Iridectomy and Iridotomy, setting forth the advantageous and what may be accomplished by each. It is prepared with care and exactness, and illustrated with several cases, and is but another evidence of the rapidly extending reputation of that distinguished oculist.

